# Care dependency in older stroke patients with comorbidities: a latent profile analysis

**DOI:** 10.3389/fnagi.2024.1366380

**Published:** 2024-05-28

**Authors:** Qinger Lin, Xiaohang Dong, Tianrong Huang, Hongzhen Zhou

**Affiliations:** ^1^Department of Nursing, Nanfang Hospital, Southern Medical University, Guangzhou, China; ^2^School of Nursing, Southern Medical University, Guangzhou, China; ^3^Department of Neurology, Nanfang Hospital Baiyun Branch, Southern Medical University, Guangzhou, China; ^4^Department of Neurology, The Third Affiliated Hospital of Southern Medical University, Guangzhou, China

**Keywords:** care dependency, latent profile analysis, stroke, comorbidity, older adult

## Abstract

**Objectives:**

To explore latent profiles of care dependency in older stroke patients with comorbidities and to analyze the factors influencing different latent profiles.

**Methods:**

A total of 312 older ischemic stroke patients with comorbidities were included in the analysis. Latent Profile Analysis (LPA) was used to classify the participants into potential subgroups with different types of care dependency. The influencing factors of the classification of care dependency subgroups were determined using multivariate Logistic regression analysis.

**Results:**

The care dependency score of older ischemic stroke patients with comorbidities was (51.35 ± 13.19), and the patients could be classified into 3 profiles, namely Universal dependency (24.0%), Moderate activity-social-learning dependency (28.0%), and Mild activity-social-learning dependency (48.0%); caregiver, BI at admission, and functional impairments were independent factors influencing care dependency (*P* < 0.05).

**Conclusion:**

There are three latent profiles of care dependency in older ischemic stroke patients with comorbidities. According to the characteristics of various populations, medical staff are able to implement specific interventions to lower the level of dependency and further improve the quality of life of patients.

## 1 Introduction

With 17.04 million strokes occurring in China among those over the age of 40 by 2019, stroke is the country’s leading cause of death (Wang, 2022). Ischemic stroke accounts for roughly 60 to 80% of all strokes and is characterized by high mortality, disability, and recurrence rates (Peng, 2018). With the improvement of medical standards, the focus has shifted from stroke mortality to post-stroke quality of life (Kapral and Bushnell, 2021). According to global surveys, stroke ranks first among 18 causes of disability, making it the leading cause of disability. Most stroke survivors experience hemiplegia, aphasia, swallowing difficulties, or other potential physical impairments, severely limiting their independent self-care performance in daily life (Murray et al., 2013).

By 2050, China is projected to have 320 million people aged over 65, with over 180 million suffering from chronic illnesses. Furthermore, it is estimated that 85% of elderly patients admitted to hospitals will have three or more illnesses (Liao and Wang, 2022). A study investigating the comorbidities among ischemic stroke patients in China has revealed that a staggering 90% of these patients suffer from at least one chronic disease, while an impressive 49.4% of them concurrently have four or more chronic diseases. Among these comorbidities, hypertension and dyslipidemia stand out as the most prevalent conditions (Hu et al., 2024). Additionally, another study has indicated that hypertension and heart disease are the most common comorbidities in patients with cerebral infarction (He et al., 2023). Overall, cardiovascular and metabolic diseases predominantly feature in the comorbidities of stroke patients. In the older age demographic, stroke is prevalent, and among ischemic stroke patients in this group, comorbidities significantly increase. Those with multiple concurrent chronic diseases face a notably higher incidence of adverse events compared to those with only one concurrent chronic disease (Hao et al., 2021; Zhang et al., 2021). The functional impairments, nutritional abnormalities, and depression resulting from stroke, along with the cumulative burden, consumption, and sense of futility associated with long-term comorbidities, contribute to a notable reduction in patients’ self-care abilities and a significant increase in care dependency (Koller et al., 2014).Thus, comorbidity is an important part of post-stroke care and that health systems need to shift their focus to developing comprehensive care programs.

Care dependency, whose concept is derived from theories of human need and self-care (Dijkstra et al., 1996, 1998). Care dependency represents a unique form of dependency. While it correlates with physiological limitations and unmet needs, it is not synonymous with these concepts. Care dependency entails subjective support and requirements to compensate for deficiencies in self-care (Boggatz et al., 2007). Stroke patients commonly experience care dependency. An investigation of 262 stroke inpatients in the older age group revealed a care dependency occurrence rate of 80.5% (Han and Zhang, 2019). Similarly, another study on the same cohort emphasized a care dependency rate of 100% (Wang et al., 2022), highlighting the significant dependency levels among stroke patients during hospitalization. At the 6-month and 1-year follow-ups, respectively, Caljouw examined changes in dependency in older residents of care institutions and noted the strong relationship between high dependency and death (Caljouw et al., 2014). Considering the significant number of stroke patients in China, it’s essential to grasp the present level of care dependency and enact essential care strategies.

Previous studies often assumed homogeneity within the sample, treating the entire sample as a homogeneous entity and estimating a set of average parameters. However, variable-centered research methods focus on the relationships between variables rather than individual differences, aiming to understand the overall patterns and associations within the data. This approach may overlook individual heterogeneity within the sample. “Person-Centered” research methods acknowledge that samples are composed of subgroups with different characteristics, and identifying these subgroups and exploring their relationships with influencing factors are important research objectives. “Person-Centered” research methods focus more on individual differences and group heterogeneity, providing support for a more detailed depiction of subgroup characteristics (Muthen and Muthen, 2000; Kim, 2014; Wang and Wang, 2023). Latent Profile Analysis (LPA) is a statistical method that embodies a “Person-Centered” approach, utilizing unobserved (latent) groups based on individuals’ responses to indicators for grouping similar people together. Unlike conventional classification techniques like cluster analysis or k-means clustering, LPA is model-based, enabling a mathematical assessment of the fidelity of the proposed LPA model to the dataset (Nylund et al., 2007; Wen et al., 2023). By utilizing LPA, our study aims to identify distinct patient subgroups based on care dependency levels, empowering healthcare providers to customize intervention strategies and effectively address the unique needs of each subgroup.

Thus, the research question (hypothesis) of our study is: “What are the latent profiles that exist among different older stroke patients with comorbidities and what factors can be used to predict the risk of higher levels of care dependency among these patients?” To address the above questions, two main processes were conducted in our study. Firstly, we attempted to utilize Latent Profile Analysis (LPA) to group 312 older stoke patients into subgroups, aiming to identify different classes of care dependency and compare the differences among them. Secondly, we explored the utility of physiological, psychological, and social factors in distinguishing between subgroups. Specifically, we aimed to identify factors that may help predict high levels of dependency, with the goal of facilitating early detection, precise targeting, and tailored interventions.

## 2 Materials and methods

### 2.1 Design and settings

#### 2.1.1 Study design

The study was a cross-sectional study in which patients with ischemic stroke who were hospitalized in two tertiary care hospitals in Guangzhou from September 2022 to June 2023 were selected using a convenience sampling method, and patients who met the inclusion criteria were invited to participate in the study by combining medical record screening and bedside condition assessment during the patients’ hospitalization.

#### 2.1.2 Participants and recruitment

The inclusion criteria were as follows: (1) age ≥ 60 years; (2) patients with first-ever ischemic stroke with a hospital stay of ≥ 5 days; (3) the primary clinical diagnosis is ischemic stroke, meeting the diagnostic criteria in “Diagnostic Highlights of Various Major Cerebrovascular Diseases in China 2019” (Zeng et al., 2019): ① acute onset of focal neurological deficits; in rare cases, it may involve global neurological deficits ② confirmation of corresponding cerebral infarct by head CT/MRI, or symptoms and signs lasting over 24 h, or causing death within 24 h ③ exclusion of non-ischemic causes; (4) Combine at least one chronic disease that aligns with the International Classification of Diseases (ICD-11); (5) stable vital signs and clear consciousness. There is some ability to communicate and understand, and the diagnosis by the physician is stable enough to participate in the study; (6) completed all assessments and have no missing data; (7) voluntary participation in this study. Exclusion criteria were as follows: (1) severe aphasia; (2) accompanied by severe cognitive impairment or mini-mental state examination (MMSE) score < 10 (Folstein et al., 1975).

#### 2.1.3 Sample size

According to the sample size calculation formula for observational studies:

N=μ2a/2⁢π⁢(1-π)δ2

According to previous studies, the rate of care dependency during hospitalization for older stroke patients is 80.5 (Han and Zhang, 2019), π = 80.5%, δ = 0.05, μ^2^_α/2_ = (1.96)^2^, *N* = 241, and a sample size of 301 was obtained considering a 20% dropout rate.

(π refers to the occurrence probability of care dependency among elderly stroke patients. α represents the type I error, which is typically set at 0.05. By looking up the *t*-value table, μ^2^_α/2_ = (1.96)^2^. δ represents the allowable error, and since there is no recognized professional standard for the allowable error of the observed indicator, we have adopted an estimation method, combining previous studies where the allowable error is often set at 0.05. We set a 20% dropout rate to account for non-response. Based on previous research, considering a dropout rate of 10 to 20% is essential for calculating the final sample size to ensure the accuracy of the study.)

### 2.2 Instrument

In our study, we have embraced a multidimensional framework that integrates various factors rooted in the biopsychosocial model. Initially formulated by Engel, this model argues that the etiology of illness encompasses not just biological factors like bacteria and viruses, but also psychological aspects such as stress, anxiety, and depression, along with social determinants like life events, employment, and economic status. In preventing diseases and fostering health, it is imperative to consider all three of these categories (Engel, 2012). Thus, in diagnosing and treating illnesses, a comprehensive approach that incorporates these diverse dimensions is warranted. To this end, we have utilized the following instruments for our study.

#### 2.2.1 General information questionnaire

Designed by the researchers themselves, it includes a demographic information questionnaire and a stroke characteristics questionnaire. It was used to collect demographic information such as patients’ gender, age, BMI, education, marital status, place of residence, caregiver, income, payment for medical care and disease-related information such as smoking, alcohol consumption, stroke site and type, the number of functional impairment and BI (Barthel Index) at admission.

#### 2.2.2 Care Dependency Scale (CDS)

The Care Dependency Scale (CDS), converts the 14 basic human needs proposed by Henderson into 15 assessment items (Dijkstra et al., 2000). In Zhang sinicized the CDS and tested its reliability in an older population (Zhang et al., 2014). The scale used the Likert 5-point scale, with scores of 1–5 representing 5 degrees from complete dependency to complete independence in order; the total score range was 15–75, with lower scores indicating a greater degree of dependence. The Cronbach’s α coefficient for the Chinese version of the Care Dependency Scale was 0.959, with inter-rater reliability KaPPa values of 0.84–0.899 and retest reliability of 0.83–0.90.

#### 2.2.3 National Institutes of Health Stroke Scale (NIHSS)

It was originally developed in 1989, the NIHSS includes the following domains: level of consciousness, eye movements, integrity of visual fields, facial movements, arm and leg muscle strength, sensation, coordination, language, speech and neglect. Item scores are summed to a total score ranging from 0 to 42 (the higher the score, the more severe the stroke) (Kwah and Diong, 2014).

#### 2.2.4 Cumulative Illness Rating Scale for the Geriatric (CIRS-G)

The CIRS-G currently consists of 14 systems, each assessed on a 0–4 scale of severity, each system is scored as follows: 0 (none), no impairment to that organ/system; 1 (mild), impairment does not interfere with normal activity; treatment may or may not be required; prognosis is excellent; 2 (moderate), impairment interferes with normal activity, treatment is needed, prognosis is good; 3 (severe), impairment is disabling, treatment is urgently needed, prognosis is guarded; 4 (extremely severe), impairment is life-threatening, treatment is urgent or of no avail; poor prognosis (Salvi et al., 2008). The Crombach’s α coefficient was 0.83, and an inter-rater reliability of 0.81.

Evaluation indicators are as follows:

The most widely used evaluation index is the TSC (total score), which is the sum of the total scores of the 14 systems, with a total score range of 0–56. In this study, patients’ CIRS scores were stratified as mild, moderate, or severe to distinguish the severity of their disease, with CIRS scores ≤ 14 being mild, 15 ≤ CIRS score ≤ 18 being moderate, and CIRS score ≥ 19 being severe (Zekry et al., 2010; Huntley et al., 2012). (2) The severity index is the mean score of the 13 items of the above CIRS-G excluding psychiatric/behavioral [severity index = total score of the 13 items of the CIRS-G excluding psychiatric (behavioral)/13] (3) comorbidity index: the number of items with a single item score ≥ 2 out of all 14 items of the CIRS-G (if 6 items score ≥ 2, the comorbidity index is 6).

#### 2.2.5 Social Support Rating Scale (SSRS)

SSRS was developed by Xiao (1994) in 1994 to detect the extent to which individuals receive psychological support in their social life and the utilization of that support. The scale has 10 entries, including three dimensions of objective support (3 items), subjective support (4 items), and utilization of social support (3 items). The higher the score, the higher the level of social support. The Crombach’s α coefficient for the scale was 0.90.

#### 2.2.6 Mini-mental state examination (MMSE)

The MMSE was first developed by Folstein, which mainly includes five aspects: orientation, memory, recall, attention and calculation, and language ability, with a total of 19 items and a total score of 30 points, and the higher the score, the better the cognitive function(Folstein et al., 1975). The Crombach’s α coefficient of the Chinese version of the scale is 0.833, and the retest reliability is 0.924. According to Chinese standards, the cut-off value of MMSE is 27, with mild cognitive impairment ranging from 21–26, moderate cognitive impairment ranging from 10 to 20, and severe cognitive impairment below 9.

#### 2.2.7 Self-Rating Depression Scale (SDS) and Self-Rating Anxiety Scale (SAS)

The SDS, developed by Zung in 1965, and the SAS developed by Zung in 1971, both contain a total of 20 items (Zung, 1965, 1971). These two scales describe the patient’s state of depression and anxiety over the past week. The cutoff score for the Chinese version of the SDS is 53 points, with mild depression ranging from 53–62 points, moderate depression from 63–72 points, and severe depression exceeding 72 points. The cutoff score for the Chinese version of the SAS is 50 points, with mild anxiety ranging from 50–59 points, moderate anxiety from 60–69 points, and severe anxiety exceeding 70 points. The Cronbach’s α coefficients for the Chinese versions of the SDS and SAS scales are 0.860 and 0.931 respectively.

### 2.3 Data collection and quality control

**FIGURE 1:**
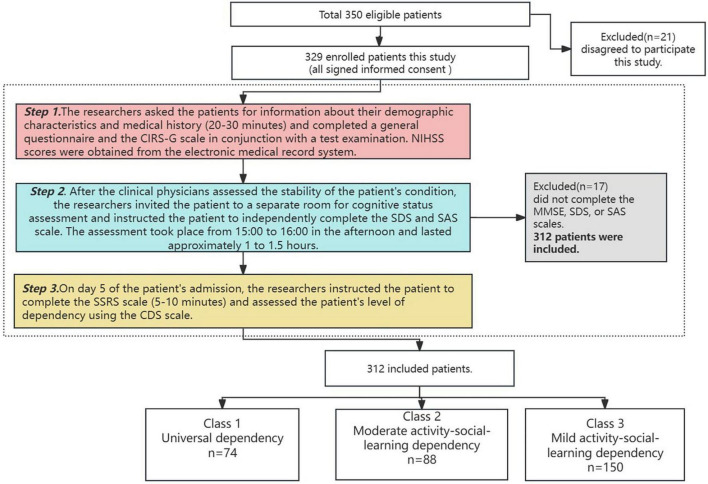
Data collection process.

The procedures for data collection were as follows:

(1) After patients were admitted to the hospital, the researcher sent invitations to patients who met the inclusion criteria and all patients signed an informed consent form. Patients were assessed by the researcher and a clinician for their overall condition, as well as asked for personal information, past medical history. In conjunction with later laboratory testing, the researcher recorded the pertinent data and performed a detailed determination of comorbidities. NIHSS scores at admission were obtained in the electronic medical record system. (2) After the patients had been in the hospital for more than five days and their conditions were stable, the researcher and a rehabilitation therapist invited the patients to a separate room to conduct a cognitive status check using the MMSE and instructed the patients to use the SDS and SAS to self-assess their depression and anxiety status. (3) The researcher scored the patient’s level of dependency using the CDS by providing medical care and instructed patients to complete the SSRS (Figure 1).

Quality control:

(1) Prior to the commencement of the study, our research team thoroughly validated the scientific and practical aspects of the research protocol and conducted a pre-survey. We also evaluated the reliability and validity of the instruments used in the study. (2) All members of the research team underwent uniform training before the study to ensure they were familiar with the assessment methods for the instruments used. Research personnel provided detailed information about the study background, objectives, and assessment procedures to participants, obtained their consent, and obtained signed informed consent forms. Participants were guided to complete the questionnaires and instruments using standardized instructions, and the completed forms were collected on the spot and checked for completeness. (3) The core instrument used in this study, the Chinese version of the CDS, was authorized by the team responsible for its translation. As it is a third-party instrument, researchers in the study team who used the CDS carefully studied the development of the original instrument, the process of translating the instrument, and relevant articles on the concept of nursing dependency, and received relevant training.

### 2.4 Statistical analysis

The data were analyzed using IBM SPSS 25.0 and Mplus 8.3.

Mplus 8.3 was selected for latent profile analysis based on the exogenous variables (CDS score as a continuous variable). Starting with a single class model, the number of class in the model was gradually increased until the best model fit indicators were achieved.

Model fitting indicators include:

(1) Akaike information criteria (AIC), Bayesian information criteria (BIC) and adjusted BIC, The smaller the value is, the better the model fits.
(2) Entropy value: the value range is 0–1, the closer to 1 means the more accurate classification.
(3) Lo-Mendell-Rubin (LMR) and Bootstrap-based likelihood ratio test (BLRT) are used for model comparison, and when the test is significant (*P* < 0.05), it means that the *k^th^* model is better than the *k*–1*^th^* model. The best-fitting model was selected by considering the above indicators in the fitting results of each type of model together.

IBM SPSS 25.0 was used for data description and analysis. The measurement data conforming to normal distribution were expressed as (M ± SD), and one-way ANOVA was used for comparison between multiple groups; the count data were expressed as number of cases and percentages, and the χ^2^ test or Fisher’s exact probability method was used for comparison between multiple groups. Multivariate logistic regression analysis was used to explore the factors influencing care dependency in older ischemic stroke patients with comorbidities, and when *P* < 0.05 indicates a statistically significant difference.

## 3 Result

### 3.1 General information of patients

During the baseline period, we invited 350 patients to participate in the study. After screening, a total of 312 patients were finally enrolled, with a response rate of 89.1% (Figure 1). The mean age of the participants, who ranged in age from 60 to 90 (66.83 ± 7.70). Among them, 67.3% were male, with a mean BMI of 23.91. Most were received middle school educated (42.9%), married (80.1%), living in urban areas (74.3%), with a caregiver as spouse (63.7%), with a mean monthly income of 3–5,000 CNY (Chinese yuan), and were covered by health insurance (85.6%). The majority of patients did not smoke or consume alcohol.

A total of 37% of patients had a stroke on the left side of the brain, while 63% of patients had a small artery occlusive stroke. On admission, 58% of patients had a BI between 46 and 99, 91.9% of patients had an NIHSS score between 0 and 7, and 51.9% had a type functional impairment. Mild cognitive impairment was present in 51.6% of patients, but most patients had no depressive or anxiety. The mean scores of CIRS-G and SSRS were 9.27 ± 3.36 and 38.97 ± 2.60, respectively.

The care dependency score for 312 patients was 51.35 ± 13.19. The items ranked lowest in score were: item 4, mobility, with a score of 2.61 ± 1.36; item 9, hazard avoidance, with a score of 2.72 ± 1.18; item 14, recreational activities, with a score of 2.77 ± 1.10; item 15, learning ability, with a score of 3.14 ± 0.96; and item 8, personal hygiene, with a score of 3.51 ± 1.39. Detailed scores for each item are listed in Table 1.

**TABLE 1:** Description and scoring of each item in the CDS (*N* = 312).

Item	Descriptions of each item	Min.	Max.	Score (M ± SD)
Total score		17	75	51.35 ± 13.19
Eating	Meeting one’s own dietary needs	1	5	4.03 ± 1.30
Incontinence	Independently managing bowel and bladder movements	1	5	4.08 ± 1.28
Body posture	Adopting appropriate body positions independently while lying down or sitting	1	5	3.96 ± 1.17
Mobility	Independently moving out of bed and walking	1	5	2.61 ± 1.36
Day/night pattern	Maintaining a normal day-night rhythm without disturbances	1	5	4.45 ± 0.89
Dressed and undresses	Independently putting on and taking off clothing	1	5	3.75 ± 1.34
Body temperature	Regulating one’s body temperature through measures such as adjusting clothing	1	5	4.08 ± 1.16
Hygiene	Completing personal hygiene tasks including washing face, brushing teeth, and combing hair	1	5	3.51 ± 1.39
Avoidance of danger	Ensuring personal safety	1	5	2.72 ± 1.18
Communication	Engaging in communication with others	1	5	4.08 ± 1.02
Contact with others	Initiating, maintaining, and terminating social interactions	1	5	3.84 ± 1.10
Sense of rules and values	Adhering to societal norms and rules independently	1	5	4.18 ± 0.99
Daily activities	Organizing daily activities independently	1	5	3.29 ± 1.21
Recreational activities	Participating in external activities independently	1	5	2.77 ± 1.10
Learning ability	Self-directed learning and acquisition of knowledge and skills.	1	5	3.14 ± 0.96

### 3.2 Latent profiles and naming of care dependency in patients

To address the primary aims of this study, we performed LPA to class the participants into latent subgroups based on their responses on the CDS.

Based on the findings of the care dependency evaluation in older ischemic stroke patients with comorbidities, five models were fitted in this study. As the number of model class increases, the values of AIC, BIC, and aBIC decrease, while LMR and BLRT values show a similar trend. The entropy values for each model are all greater than 0.9, indicating a good fit of the models. Since the LMR values for the five-model are not significant, we focused our discussion on the first four models. The entropy value for the four-model is lower than that of the three-model, indicating higher classification accuracy for the three-model. Although the entropy value for the three-model is smaller than that of the two-model, both the LMR and BLRT values are significant, indicating a better fit for the three-model than the two-model, and the probabilities of each class in the three-model are greater than 5%. Considering both fit indices and practical significance, we select model three, which contains three latent profile, as the best-fitting model. As shown in Table 2.

**TABLE 2:** Modle fit indices for latent profile analysis

No. of profiles	AIC	BIC	aBIC	Entropy	LMR	BLRT	Latent profile proportion (%)
1	14659.159	14771.449	14676.299	–	–	–	–
2	11894.388	12066.566	11920.670	0.976	*P* = < 0.001	*P* = <0.001	37/63
**3**	**11021.059**	**11253.125**	**11056.482**	**0.972**	*P* = **0.004**	*P* = ** < 0.001**	**24/28/48**
4	10713.260	11005.214	10757.824	0.952	*P* = 0.453	*P* = < 0.001	25/25/10/40
5	10464.490	10816.333	10518.196	0.953	*P* = 0.298	*P* = < 0.001	8/28/18/15/31

AIC, Akaike information criteria; BIC, Bayesian information criteria; LMR, Lo-Mendell-Rubin; BLRT, Bootstrap-based likelihood ratio test. Bold values indicate that the model in that class is the best model we have chosen.

**FIGURE 2:**
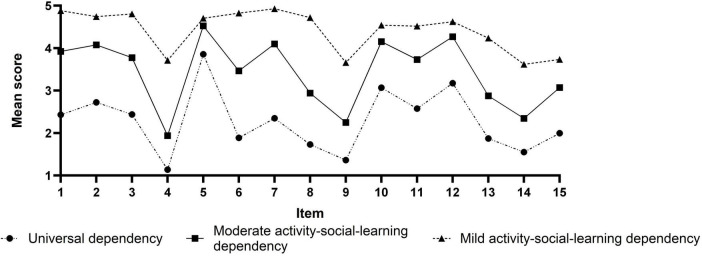
Characteristic distribution of three latent profiles.

Based on three profiles model, the mean scores of each profile in the 15 items of care dependency are shown in Figure 2. Class 1, class 2, and class 3 were named according to their characteristic distributions. Class 1 was labeled “Universal dependency” and had low scores, indicating that these patients’ overall levels of dependency were high, accounting for 24.0% (74/312); the Class 2 had moderately high scores, but item 4 (activity), item 9 (avoidance of danger), and item 13–15 (daily activities, recreation, and learning) had low scores, and were named Moderate activity-social-learning dependency, accounting for 28.0% (88/312); the Class 3 had generally high scores, but item 4, item 9, and item 14–15 still had slightly low scores, and were named Mild activity-social-learning dependency, accounting for 48.0% (150/312).

### 3.3 A univariate analysis of latent profiles of care dependency in older stroke patients with comorbidities

The differences were statistically significant (*P* < 0.05) when comparing the caregiver, BI score at admission, stroke site, stroke type, type of functional impairment, NIHSS score at admission, MMSE score, SDS score, CIRS-G score, and SSRS score among the three profiles. The differences were not statistically significant (*P* > 0.05) when the remaining variables were compared. As shown in Tables 3-1, 3-2.

**TABLE 3-1:** Results of a univariate analysis of latent profiles of care dependency in older ischemic stroke patients with comorbidities (*N* = 312).

	Total *N* (%)	Class 1 (*n* = 74)	Class 2 (*n* = 88)	Class 3 (*n* = 150)	F/χ^2^	*P*
**Gender**						
Man (%)	210 (67.3)	48 (64.9)	67 (76.1)	95(63.3)	4.39	0.109
woman (%)	120 (32.0)	26 (35.1)	21 (23.9)	55(36.7)		
Age (M ± SD)	66.83 ± 7.70	67.89 ± 8.03	67.00 ± 8.15	66.06 ± 7.09	1.76	0.171
BMI (M ± SD)	23.91 ± 3.23	23.86 ± 3.72	23.89 ± 2.86	23.95 ± 3.18	0.02	0.968
**Education**						
Elementary school and below (%)	108 (34.6)	27 (36.5)	26 (29.5)	55(36.7)	5.63	0.469
middle School (%)	134 (42.9)	36 (48.6)	37 (42)	61(40.7)		
high School (%)	38 (12.2)	6 (8.1)	15 (17)	17(11.3)		
college and above (%)	32 (9.3)	5 (6.8)	10 (11.4)	17(11.3)		
**Marriage**						
Married (%)	250 (80.1)	55 (74.3)	71 (80.7)	124(82.7)	2.98(FISH)	0.550
Widowed (%)	57 (18.2)	18 (24.3)	15 (17)	24(16)		
Divorced (%)	5 (1.7)	1 (1.4)	2 (2.3)	2(1.3)		
**Location**						
Urban areas (%)	232 (74.3)	56 (75.7)	66 (75)	110(73.3)	0.17	0.934
Rural areas (%)	80 (25.7)	18 (24.3)	22 (25)	40(26.7)		
**Caregiver**						
Spouse (%)	199 (63.7)	37 (50)	51 (58)	111(74)	26.70	< 0.001
Adult children (%)	99 (31.7)	35 (47.3)	31 (35.2)	33(22)		
Other relatives (%)	14 (4.6)	2 (2.8)	6 (6.8)	6(4)		
**Income**						
< 3k (%)	1 (0.5)	0	0	1(0.7)	2.56	0.926
3–5k (%)	226 (72.4)	52 (70.3)	66 (75)	108(72)		
5–7k (%)	53 (16.9)	14 (18.9)	12 (13.6)	27(18)		
> 7k (%)	32 (10.2)	8 (10.8)	10 (11.4)	14(9.3)		
**Pay**						
Self-financed (%)	45 (14.4)	8 (10.8)	13 (14.8)	24(16)	4.31	0.310
Medical Insurance (%)	267 (85.6)	66 (89.2)	73 (85.2)	126(84)		
**Smoke**						
No (%)	176 (56.4)	47(63.5)	43 (48.9)	86(57.3)	3.61	0.170
Yes (%)	136 (43.6)	27(36.5)	45 (51.1)	64(42.7)		
**Alcohol**						
No (%)	254 (81.4)	66 (89.2)	73 (83)	115(76.7)	9.56	0.140
Yes (%)	58 (18.6)	8 (10.8)	15 (17)	35(23.3)		

**TABLE 3-2:** Results of a univariate analysis of latent profiles of care dependency in older ischemic stroke patients with comorbidities (*N* = 312).

	Total *N* (%)	Class 1 (*n* = 74)	Class 2 (*n* = 88)	Class 3 (*n* = 150)	F/χ^2^	*P*
**Stroke site**						
Left side of the brain (%)	116 (37.1)	26 (35.1)	28 (31.8)	62 (41.3)		
Right side of the brain (%)	110 (35.2)	31 (41.9)	35 (39.8)	44 (29.3)	17.88	0.022
Cerebellum (%)	14 (4.5)	3 (4.1)	3 (3.4)	8 (5.3)		
Brainstem (%)	41 (13.1)	12 (10.2)	14 (15.9)	15 (10.0)		
Other (%)	31 (10.1)	2 (2.7)	8 (9.1)	21 (14.1)		
**Stroke type**						
Large Artery atherosclerosis(%)	103 (33.0)	44 (59.5)	31 (35.2)	28 (18.7)		
Cerebral embolism	5 (1.6)	3 (4.1)	2 (2.3)	0	55.14	< 0.001
Small artery occlusive (%)	195 (63)	25 (33.8)	54 (61.4)	116 (77.3)		
Cerebral Watershed Infarction (%)	6 (1.9)	0	1 (1.1)	5 (3.3)		
Hemorrhagic cerebral Infarction (%)	2 (1.0)	2 (2.7)	0	0		
Other (%)	1 (0.5)	0	0	1 (0.7)		
**NIHSS score**						
≤ 7 (%)	287 (91.9)	56 (75.7)	81 (92.0)	150 (100.0)	39.78	< 0.001
8–16 (%)	25 (8.1)	18 (24.3)	7 (8.0)	0		
**Functional impairment**						
None (%)	116 (37.1)	3 (4.1)	19 (21.6)	94 (62.7)		
One (%)	162 (51.9)	52 (70.3)	62 (70.4)	48 (32.0)	112.40	< 0.001
Two or more (%)	34 (10.0)	19 (25.6)	7 (8.0)	8 (5.3)		
**BI at admission**						
0–45 (%)	94 (30.1)	61 (82.4)	30 (34.1)	3 (2.0)		
46–99 (%)	181 (58.0)	13 (17.6)	57 (64.8)	111 (74.0)	171.16	< 0.001
100(%)	37 (11.9)	0	1 (1.1)	36 (24.0)		
**Cognition (MMSE)**						
Severe (%)	6 (1.9)	3 (4.1)	2 (2.3)	1 (0.7)		
Moderate (%)	161 (51.6)	54 (73.0)	49 (55.7)	58 (38.7)	35.52	< 0.001
Mild (%)	111 (35.6)	13 (17.6)	33 (37.5)	65 (43.3)		
Normal (%)	34 (10.9)	4 (5.4)	4 (4.5)	26 (17.3)		
**Depression (SDS)**						
Normal (%)	248 (79.4)	51 (68.9)	74 (84.1)	123 (82.0)		
Mild (%)	49 (15.7)	17 (23.0)	9 (10.2)	23 (15.3)	10.60	0.040
Moderate (%)	14 (4.5)	5 (6.8)	5 (5.7)	4 (2.7)		
Severe (%)	1 (0.4)	1 (1.3)	0	0		
**Anxiety (SAS)**						
Normal (%)	225 (72.1)	49 (66.2)	68 (77.3)	108 (72.0)		
Mild (%)	65 (20.8)	14 (18.9)	18 (20.5)	33 (22.0)	9.66	0.090
Moderate (%)	20 (6.4)	10 (13.5)	2 (2.3)	8 (5.3)		
Severe (%)	2 (0.7)	1 (1.4)	0	1 (0.7)		
CIRS-G (M ± SD)	9.27 ± 3.36	11.20 ± 3.79	9.41 ± 3.22	8.23 ± 2.75	22.17	< 0.001
SSRS (M ± SD)	38.97 ± 2.60	38.53 ± 2.85	38.64 ± 2.57	39.38 ± 2.46	3.70	0.020

M, mean; SD, standard deviation; F, One-way ANOVA; χ^2^, Chi-square; H: Class 1, universal dependency; Class 2, moderate activity-social-learning dependency; Class 3, mild activity-social-learning dependency.

### 3.4 Multivariate logistic regression analysis of factors influencing care dependency scores in older ischemic stroke patients with comorbidities

A multivariate logistic regression analysis was performed using the care dependency score of older ischemic stroke patients as the dependent variable and the indicators with statistically significant differences in the univariate analysis as the independent variables. The variable assignments are as follows: caregivers, 1 = spouse, 2 = adult-child, 3 = other relatives; stroke type, 1 = Large artery atherosclerosis,2 = Small artery occlusive, 3 = other; stroke site, 1 = left, 2 = right, 3 = brainstem, 4 = other; BI at admission, 1 = score 0–45, 2 = score 46–99, 3 = score 100; functional impairment, 0 = none, 1 = one, 2 = two or more; NIHSS score, 1 = ≤ 7, 2 = 8–16 (Kim et al., 2010); MMSE score, 0 = > 27, 1 = 21–26, 2 = 10–20, 3 = < 9; SDS score, 0 = normal, 1 = mild, 2 = moderate, 3 = severe; SAS score, 0 = normal, 1 = mild,2 = moderate, 3 = severe; CIRS-G, SSRS score are entered as measured values. Class 1 is used as the control group. In the case of categorical variables, the last assigned variable is used as the dummy variable.

The results indicate that the multiple logistic regression model has a goodness-of-fit chi-square of 527.992, a pseudo *R*^2^ of 0.559, and a likelihood ratio chi-square of 255.515, with a *p*-value less than 0.001. The primary caregiver, the Barthel Index (BI) at admission, and functional impairment are independent influencing factors across different profiles.

Specifically, In comparison to the “Mild activity-social-learning dependency” (C3), the factors influencing elderly ischemic stroke comorbid patients to be classified into the “Universal dependency” (C1) are the primary caregiver’s role and residual functional impairment. Being cared for by adult children reduces the likelihood of being classified into the C1 compared to the C3 by 0.29 times (OR = 0.290, 95% CI: 0.109, 0.769), while having no residual functional impairment decreases the probability of being classified into the C1 compared to the C3 by 0.132 times (OR = 0.132, 95% CI: 0.018, 0.973). Therefore, being cared for by other relatives and having one or more functional impairment are more likely to result in classification into the “universal dependency”.

In comparison to the “Mild activity-social-learning dependency” (C3), the factors influencing elderly ischemic stroke comorbid patients to be classified into the “Moderate activity-social-learning dependency” (C2) are the primary caregiver’s role and the admission Barthel Index (BI) score. Being cared for by children decreases the probability of being classified into the (C2) compared to the (C3) by 0.439 times (OR = 0.439, 95% CI: 0.211, 0.913); having an admission BI score of 0–45 and 46–99 increases the likelihood of being classified into the (C2) compared to the (C3) by 164.346 times (OR = 164.346, 95% CI: 14.328, 88.097) and 9.703 times (OR = 9.703, 95% CI: 1.217, 77.361), respectively. Therefore, having a caregiver who is not a child and a lower BI score makes it more likely to fall into the “moderate” group. As shown in Table 4.

**TABLE 4:** Multivariate logistic regression analysis of factors influencing different latent profiles of patients’ care dependency.

Dependent variable	Independent variable		β	SE	Wald χ^2^	*p*-value	OR (95% CI)
Class 1	Caregiver	Adult child	−1.24	0.50	6.19	0.013	0.290 (0.109–0.769)
		Other relative (refer)					
	Functional impairment	None	−2.02	1.02	3.95	0.047	0.132 (0.018–0.973)
		Two or more (refer)					
Class 2	Caregiver	adult child	−0.74	0.36	4.20	0.041	0.478 (0.236–0.969)
		Other relative (refer)					
	BI at admission	0–45	5.10	1.25	16.80	< 0.001	164.346 (14.328–188.097)
		46–99	2.27	1.06	4.60	0.032	9.703 (1.217–77.361)
		100 (refer)					

Only statistically significant variables are listed (*P* < 0.05). Multiple categorical variables are encoded using the last assigned variable as the dummy variable. Class 3 as control; Class1, universal dependency; Class 2, moderate activity-social-learning dependency; Class 3, mild activity-social-learning dependency.

### 3.5 Comorbidity of different profiles of older ischemic stroke patients

The top 7 organs (systems) overall were hypertension (81%), blood vascular system (50%), endocrine and metabolic system (40%), respiratory system (39%), cardiac system (20%), psychiatric system (17%), and musculoskeletal system (10%), as shown in Table 5. According to the classification criteria of the first assessment indicator (TSC) of the CIRS-G scale, patients can be divided into three groups: mild comorbidity (score 0–14), 276 patients (88%); moderate comorbidity (score 15–18), 27 patients (9%); and severe co-morbidity (score > 19), 9 patients (3%); the gender, comorbidity index, severe index, and NIHSS score at admission were compared, and statistically significant differences (*P* < 0.05) were found, while the other variables were not significant, as shown in Table 6.

**TABLE 5:** The first 7 organs (systems).

Organ (system)	Class 1	Class 2	Class 3	*N* (%)	Chi-square	*P*
Hypertension	68	72	114	254(81%)	8.28	0.016
Vascular	46	41	70	157(50%)	5.44	0.066
Endocrine-Metabolic	36	40	50	126(40%)	6.14	0.047
Respiratory	32	38	53	123(39%)	2.02	0.360
Cardiac	29	21	15	65(20%)	26.28	< 0.001
Psychiatric	18	19	18	55(17%)	6.51	0.039
Musculoskeletal	8	7	17	32(10%)	0.72	0.698

**TABLE 6:** Comparative results of relevant parameters in patients with different CIRS-G scores.

	Mild *n* = 276(88%)	Moderate *n* = 27(9%)	Severe *n* = 9(3%)	F/χ^2^	P
Gender man	178	25	7	9.28	0.010
Woman	98	2	2		
Class 1	34.45 ± 7.12	36.29 ± 6.26	26.50 ± 6.36	1.509	0.298
Class 2	52.40 ± 4.71	50.00 ± 5.09	47.50 ± 2.12	1.98	0.141
Class 3	66.96 ± 4.14	67.42 ± 3.70	70.00 ± 3.46	0.86	0.426
Age	65.74 ± 8.94	65.78 ± 7.18	71.56 ± 8.23	1.91	0.147
Comorbidity index	3.12 ± 0.93	4.74 ± 0.76	6.11 ± 0.78	80.89	< 0.001
Severity index	0.62 ± 0.16	1.08 ± 0.08	1.35 ± 0.07	185.49	< 0.001
NIHSS score	3.00 ± 2.48	4.30 ± 2.69	6.44 ± 3.61	10.74	< 0.001

Mild, score 0–14; moderate, score 15–18; severe, score > 19. *F*, One-way ANOVA; χ^2^ Chi-square.

## 4 Discussion

### 4.1 The overall care dependency of patients

We investigated 312 hospitalized patients with comorbidities of stroke, with a CDS score of 51.35 ± 13.19, indicating a partial dependency level. Lower scores in activities such as mobility, avoidance of danger, and recreational activities align with previous research findings. This could be due to factors such as poorly controlled conditions from comorbidities like hypertension, heart disease, or diabetes, leading to symptoms such as dizziness and weakness in limbs among stroke patients, fostering a greater reliance on others due to fears of falling or being bedridden. Some patients may reduce their activities and outings to lower the incidence of adverse events. This emphasizes the need for healthcare providers to address not only patients’ physiological functioning but also prioritize their safety risks.

### 4.2 Patient can be divided into three latent profiles

We observed that care dependency among patients can be classified into three distinct profiles: Universal dependency, Moderate activity-social-learning dependency, and Mild activity-social-learning dependency. The CDS scores for these profiles are 32.09 ± 6.53, 48.41 ± 4.40, and 62.58 ± 3.87, respectively, indicating a population heterogeneity in care dependency among older ischemic stroke patients with comorbidities.

The ‘Universal dependency’ accounts for 24% of all patients (74/312). These patients scored significantly lower than the average across all items, indicating a substantial reliance on physiological, psychological, and external support. Univariate analysis revealed that compared to the other two class, these patients were more likely to have stroke affecting the dominant cerebral hemisphere and comorbid large artery atherosclerosis, with higher NIHSS scores indicating a more severe stroke. Due to the sudden onset of acute cerebrovascular events, these patients may not have had the opportunity to comprehend their condition or respond (Han and Zhang, 2019). Healthcare providers should pay special attention to changes in these patients’ conditions, remain vigilant for further deterioration in the early stages of illness, and consider employing a comprehensive compensatory care approach for this patient population.

The “Moderate activity−Social−Learning dependency” accounts for 28% of all patients (88/312). These patients scored moderately across all items, but still exhibit significant functional, social, and learning impairments. This indicates that, due to their physical and psychological characteristics, as well as disease features, some care needs of these patients have not been fully met. Healthcare providers should implement proactive and effective care measures to assist patients in regaining independence as early as possible. Once stabilized, early rehabilitation training should be initiated (Sun et al., 2023).

The “Mild activity−Social−Learning dependency” class accounts for 48% of all patients (150/312). These patients score high on all items, indicating a lower level of dependency and mild severity of illness. As their treatment progresses and their condition stabilizes, they gradually recover. It’s important to encourage these patients to participate in managing their own health, understand their condition, and prepare for discharge, including making necessary lifestyle and dietary changes to prevent relapse (Li et al., 2022).

### 4.3 The factors influencing the care dependency profiles

#### 4.3.1 Caregiver

Family members are the most important caregivers of stroke patients (Tyagi et al., 2021). In our study, we counted the number of caregiver roles in three categories: spouse, adult children, and other relatives. Spousal caregivers were the most common type of caregiver for older stroke patients. However, the results of the regression analysis in our study indicate that for patients with more severe conditions and moderate to severe dependency, having adult children as caregivers has a significant impact on reducing the patients’ dependency compared to elderly spouse or other relatives caregivers. The reasons for this may be two fold. On the one hand, for patients with severe stroke and concurrent mobility impairment, cognitive impairment and other neurological deficits, being an adult child shows a more active role in monitoring changes in the patient’s condition, improving communication with the health care team and helping the patient in making medical decisions during the early stages of the illness (van Ryn et al., 2011; Shin et al., 2013; Isenberg et al., 2018; Fenton et al., 2022). A study in Singapore noted that by adult children taking turns in care, pooling resources, and distributive support (determining which part of care each person is competent in), patients avoid to some extent the influence brought about by medical decision-making activities and become less dependent (Tyagi et al., 2021); on the other hand, it has been noted that the cultural context influences the tendency for children to care for their parents, which has an impact on the children’s own lives and work. In this regard, parents feel guilty and try to reduce their needs to ease the burden of their children (Liu et al., 2020b; Liu, 2021; Nursiswati et al., 2022), resulting in an apparent “low dependency”.

At the same time, there were differences in social support among the three profile. The satisfaction level of elderly ischemic stroke patients in our study was moderately high, which is consistent with previous research findings (Sun et al., 2023). The current changes in societal dynamics and demographic structures are reshaping family compositions and the scale of older populations. Some older individuals are experiencing prolonged separation from their adult children, resulting in a lack of alternative emotional support channels (Moonesar et al., 2016). With advancing age, physical mobility becomes constrained, and social circles shrink, leaving them with limited sources of social support beyond their children (Kong et al., 2019). A robust social support system for older adults signifies increased opportunities for better social security benefits, improved healthcare accessibility, greater resources, and enhanced social interactions. Moreover, through social networks, older adults can cultivate essential social companionship, instrumental assistance, and emotional support to mitigate negative emotions and bolster their ability to cope with illnesses (Dai et al., 2016; Bustamante et al., 2018). Although the level of social support did not exhibit statistical significance in the regression equation, the strong association between support systems and caregivers warrants further exploration in subsequent studies.

#### 4.3.2 Functional impairment

From the univariate analysis, differences are observed in residual functional impairments, cognitive status, and depression-anxiety states among the three profiles. Compared to the mild group, patients with universal and moderate group tend to have lesions concentrated in the dominant hemisphere (language areas, writing, and reading). Additionally, stroke types are mostly of the large artery atherosclerosis type and often involve more than one functional impairment.

The large-artery atherosclerosis subtype is the most common subtype of ischemic stroke, accounting for 33% of cases in Asian populations (Ornello et al., 2018). Compared to other stroke types, large artery atherosclerosis strokes pose the highest risk of recurrence and mortality, severely impacting daily activities due to post-stroke motor impairments (Osoba et al., 2019; Wu et al., 2021). In addition to age-related physiological changes and musculoskeletal degeneration, which diminish the body’s response to external pressures, neurological damage hampers mobility and balance (Wu et al., 2023). The loss of physical control and alterations in body perception often leave most first-time stroke survivors feeling disoriented, leading to a decrease in their self-care capabilities.

Several studies have indicated that the likelihood of cognitive impairment and depression is higher when stroke lesions occur in the left hemisphere compared to the right hemisphere (Broderick et al., 2014; Huang et al., 2017). This could be attributed to the anatomical and physiological functions of the hemispheres; there are extensive interconnection fibers between the frontal and temporal lobes, which are associated with memory, judgment, abstract thinking, emotions, and impulsive behavior, particularly lesions in the frontal pole area, which often manifest as mental disorders (Zhang, 2019). Additionally, research suggests that the number of functional impairments is correlated with depressive states, possibly because these impairments affect the patient’s self-esteem and induce social stigma (Van Ness et al., 2019). Cognitive impairment and depressive states can directly impact a patient’s social interaction and communication. Inadequate coping resources in the psychological and social domains may lead elderly individuals to lose interest in physical and social activities, thereby resulting in further functional decline and potentially increasing the occurrence of adverse events (Liu et al., 2020b).

#### 4.3.3 Comorbidity

The comorbidity rate of ischemic stroke is high and affects post-stroke survival (Hao et al., 2021). According to the research results, the top five comorbidities of ischemic stroke in our study are mainly related to the cardiovascular system, endocrine and metabolic system, and pulmonary diseases, which may be related to similar disease mechanisms or exposure to risk factors, such as hypertension and diabetes, which can increase vulnerability by impairing metabolic balance and cardiovascular function (Angulo et al., 2016; Cai et al., 2023a). Hypertension is the most prevalent comorbid chronic disease, and changes in blood pressure are closely related to cerebral blood supply in stroke patients. Elevated blood pressure can lead to insufficient cerebral blood flow, and abnormal blood pressure may even increase the risk of secondary intracerebral hemorrhage in ischemic stroke (Liu et al., 2020a; Cai et al., 2023b).

The severity of comorbid conditions significantly impacts stroke rehabilitation. The coexistence of multiple chronic diseases is highly correlated with dependency, particularly evident in neurological disorders (Koller et al., 2014). Compared to patients without other chronic conditions, the quality of life decrement index in comorbid individuals increases with the number of combined diseases (Li, 2021). We also found that, when comparing different genders, there are differences in the presentation of severity and comorbidity indices, and gender differences in comorbidity conditions have been mentioned in previous studies (Yan et al., 2020; Zeng et al., 2021; Basso et al., 2022). We have found that, in this study, although different degrees of comorbidities didn’t exhibit statistical significance across the three profile categories, we will continue to explore the impact of comorbidity status on patients’ long-term dependency in further longitudinal studies.

## 5 Conclusion and limitation

Our study conducted a thorough investigation into care dependency levels of 312 older stroke patients with comorbidities. The results revealed significant heterogeneity among this patient group, which could be clearly categorized into three distinct latent profiles. Furthermore, we explored the potential factors that may influence these profile classifications and identified the caregiver’s role, the presence of functional impairment, and the BI (Barthel Index) as independent and significant factors influencing the profiles

Our study had several limitations that should be acknowledged. Initially, we utilized convenience sampling, a non-probability sampling technique frequently employed in cross-sectional studies. Given the constraints of inclusion/exclusion criteria and ethical principles, convenience sampling enabled us to conduct our study with greater ease and feasibility. However, we are cognizant of the potential for selection and information bias inherent in this approach, which serves as a limitation of our study. To mitigate this, we implemented various measures throughout the research process. A portion of the questionnaires and scales were completed by the patients themselves, and while we eliminated unqualified responses during quality control, the risk of reporting bias persists. Furthermore, Our study was only conducted in two tertiary hospitals, and the relatively small sample size may reduce the representativeness of the sample. In the future, we aim to expand our sample size to validate the applicability of the identified profiles.

## Data availability statement

The raw data supporting the conclusions of this article will be made available by the authors, without undue reservation.

## Ethics statement

The studies involving humans were approved by Ethics Committee of Nanfang Hospital of Southern Medical University. The studies were conducted in accordance with the local legislation and institutional requirements. The participants provided their written informed consent to participate in this study.

## Author contributions

QL: Conceptualization, Data curation, Investigation, Methodology, Software, Writing−original draft, Writing−review and editing. XD: Data curation, Formal analysis, Investigation, Software, Writing−review and editing. TH: Data curation, Investigation, Software, Writing−review and editing. HZ: Conceptualization, Data curation, Investigation, Methodology, Software, Supervision, Writing−original draft, Writing−review and editing.
